# Complement and the atypical hemolytic uremic syndrome in children

**DOI:** 10.1007/s00467-008-0872-4

**Published:** 2008-11-01

**Authors:** Chantal Loirat, Marina Noris, Véronique Fremeaux-Bacchi

**Affiliations:** 1 Assistance Publique - Hôpitaux de Paris, Université Paris 7, Hôpital Robert Debré, Pediatric Nephrology, Paris, France; 2 Clinical Research Center for Rare Diseases Aldo e Cele Dacco, Mario Negri Institute for Pharmacological Research, Ranica, Italy; 3 Assistance Publique - Hôpitaux de Paris, Hôpital Européen Georges Pompidou, Biological Immunology Department, Paris, France; 4 Service de Néphrologie, Hôpital Robert Debré, 48 Boulevard Sérurier, 75019 Paris, France

**Keywords:** Hemolytic uremic syndrome, Alternative pathway of complement, C3, Complement factor H, Factor I, Factor B, Membrane cofactor protein, Plasma infusion, Plasma exchange, Transplantation

## Abstract

Over the past decade, atypical hemolytic uremic syndrome (aHUS) has been demonstrated to be a disorder of the regulation of the complement alternative pathway. Among approximately 200 children with the disease, reported in the literature, 50% had mutations of the complement regulatory proteins factor H, membrane cofactor protein (MCP) or factor I. Mutations in factor B and C3 have also been reported recently. In addition, 10% of children have factor H dysfunction due to anti-factor H antibodies. Early age at onset appears as characteristic of factor H and factor I mutated patients, while MCP-associated HUS is not observed before age 1 year. Low C3 level may occur in patients with factor H and factor I mutation, while C3 level is generally normal in MCP-mutated patients. Normal plasma factor H and factor I levels do not preclude the presence of a mutation in these genes. The worst prognosis is for factor H-mutated patients, as 60% die or reach end-stage renal disease (ESRD) within the first year after onset of the disease. Patients with mutations in MCP have a relapsing course, but no patient has ever reached ESRD in the first year of the disease. Half of the patients with factor I mutations have a rapid evolution to ESRD, but half recover. Early intensive plasmatherapy appears to have a beneficial effect, except in MCP-mutated patients. There is a high risk of graft loss for HUS recurrence or thrombosis in all groups except the MCP-mutated group. Recent success of liver–kidney transplantation combined with plasmatherapy opens this option for patients with mutations of factors synthesized in the liver. New therapies such as factor H concentrate or complement inhibitors offer hope for the future.

## Introduction

Hemolytic uremic syndrome (HUS) is characterized by the triad of hemolytic anemia with fragmented erythrocytes, thrombocytopenia and acute renal failure. The underlying histological lesion is thrombotic microangiopathy. In children, the most frequent form (90% of patients), called typical or post-diarrheal (D+) HUS, is caused by infection with Shiga-toxin (Stx)-producing *Escherichia coli* (STEC) [1]. It occurs mainly in children 6 months to 3 years of age, and has a relatively favorable outcome, as rapid progression to end-stage renal failure (ESRF) is exceptional and 75% of patients make long-term full recovery [1–3]. The other form, called atypical HUS (aHUS), (10% of children), occurs at any age, may be sporadic or familial, and has a poor prognosis, as approximately 50% of patients progress to end-stage renal disease (ESRD) [3].

During the past 10 years, a clear link has been demonstrated between aHUS and genetic abnormalities in complement regulatory genes. Several studies have confirmed genetic predisposition both in familial and sporadic cases, involving factors implicated in the regulation of the alternative pathway of the complement system (factor H (CFH) [4–11], CD46 (or MCP for membrane cofactor protein) [12–14], factor I (CFI) [15–17], and more recently factor B (CFB) [18] and C3 [19]), which are implicated in the formation of the alternative C3-convertase. In addition, acquired cases of aHUS associated with CFH dysfunction due to anti-CFH autoantibodies have been identified [20–22].

The aim of this review is to emphasize how progress in the understanding of the pathophysiology of aHUS has led to improved care of children with aHUS. This review also addresses which investigations should be performed, phenotype–genotype correlations, outcome and treatment. Any child with aHUS should be thoroughly investigated, since accurate diagnosis can affect the therapy of aHUS.

## HUS due to defective complement regulation

### Complement and its regulation

**Fig. 1:**
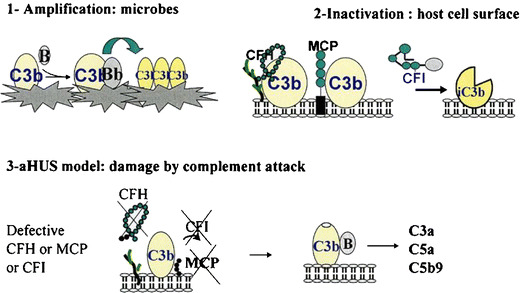
Complement activation and control. aHUS is a disease resulting from inefficient protection of the surfaces of the host’s endothelial cells in the setting of complement activation. (1) Activation of complement and covalent attachment of complement C3 to the microbial surfaces. C3b binds CFB, inducing formation of the alternative C3 convertase (C3bBb) and amplification of the C3 cleavage. (2) Protection of self cell surfaces. Regulation of the cleavage of C3 is critical. Under normal conditions the formation of C3 convertase is tightly controlled by CFH, MCP, and CFI. (3) In the case of aHUS, activation is uncontrolled and C3 convertase is formed, resulting in formation of inflammatory mediators. CFH does not attach to surfaces through its heparin/anionic-binding sites, and, thus, CFB binds C3b. Degradation of C3b to iC3b is defective in the absence of CFI and its cofactors (CFH and MCP) (from [24], with permission of the authors and Wiley–Blackwell Publishing)

Complement is an efficient and rapidly responsive component of the innate immune system and underlies one of the main effector mechanisms of antibody-mediated immunity. It has three over-arching physiologic activities: defense against pyogenic bacterial infection, the bridging of innate and adaptive immunity, and the production of anaphylatoxins. Complement is activated by three pathways: the classical pathway, the lectin pathway and the alternative pathway. These three pathways converge at the point of cleavage of C3. Although the activation of the classical and lectin pathways occurs after binding to immune complexes or microorganisms, respectively, the alternative pathway is continually activated and generates C3b, which binds indiscriminately to pathogens and host cells. On a foreign surface, i.e. a bacterium, C3b binds the CFB, which is then cleaved by factor D to form the C3 convertase C3bBb, providing exponential cleavage of C3b, and formation of C5 convertase and of a lytic membrane attack complex. The host cells are protected from the formation of C3 convertase on their surface by soluble and membrane-associated complement regulatory proteins [23] (Fig. 1) [24].

Mainly synthesized by the liver, CFH is a single-chain serum glycoprotein of 150 kDa and is the most important protein in the regulation of the alternative pathway in serum. CFH inhibits the formation of the alternative C3-convertase and accelerates its decay. CFH and CD46 serve as cofactors for the CFI, which is a serine protease. Both genes encoding CFH and CD46 are localized on the long arm of chromosome 1 at 1q32, a locus called regulators of complement activation (RCA) which contains genes encoding different regulatory proteins of complement activation. These proteins are characterized by the presence of a modular structure consisting of a tandem array of homologous units of approximately 60 amino acid residues, each called a short consensus repeat (SCR), and by their capacity to bind C3b, or C3 cleavage fragments.

### aHUS is associated with mutations in complement regulatory proteins

Presently, approximately 50% of aHUS patients have been demonstrated to have mutations in the genes of complement components and regulators (CFH, CFB, CFI, MCP and C3) and 10% of childhood cases to have CFH autoantibodies, highly suggesting that impaired control of the activity of the complement amplification convertase C3bBb is the predominant factor predisposing individuals to the disease. The year 2007 saw publication of a report on a transgenic mouse model that spontaneously develops HUS, resulting from CFH knock out (CFH −/− mice develop membranoproliferative glomerulonephritis but not HUS) with knock in of a modified CFH that carries a deletion in SCR 16–20 [25]. In this mouse model, CFH regulates C3 activation in the plasma but fails to bind to endothelial cells, similar to mutated CFH of aHUS patients, thus highlighting the dissociation of CFH metabolism and action in the plasma and on the cell surface.

#### CFH

Since the report by Thompson and Winterborn [26], several observations of complete or partial quantitative CFH deficiencies have been reported in aHUS patients, all with low serum C3 levels and normal C4 levels [27]. In 1998, Warwicker et al. reported linkage of aHUS to a locus within the RCA gene cluster containing the *CFH* gene, by genetic study of three large kindreds exhibiting no evidence of quantitative CFH deficiency and normal C3 levels [11]. They also found, in one of the families, a heterozygous nucleotide substitution leading to the change of an amino acid in the SCR 20. Additional genetic studies have also found several different heterozygous missense mutations within SCR 16 to 20 [8, 9]. At present, over 100 distinct *CFH* mutations have been reported in aHUS patients [28]. All reported mutations were heterozygous, except in 15 patients (mostly from consanguineous families) with homozygous CFH deficiency (review in [29]). The majority of mutations published up to now are located within the C-terminal domain of the protein, particularly in SCR 20. This induces a reduced ability of CFH to bind to surface-bound C3b and to the polyanions of the endothelial cells and, thus, impairs the function of the CFH protein without modifying its plasma level [7, 30]. As the majority of mutations are heterozygous, it appears that 50% of normal CFH is not sufficient to counteract the dysfunction of the mutated CFH. A hybrid *CFH-CFH R1* gene, due to either gene conversion [31] or non-homologous recombination between *CFH* and the gene of CFH-R1, has similar functional consequences [10]. Fewer than 30% of mutations have been associated with a quantitative CFH deficiency as defined by antigenic plasma levels below half of normal [6].

**Fig. 2:**
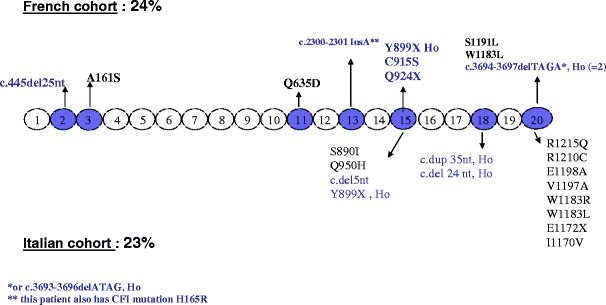
*CFH* mutations in the French (*upper line*) and Italian (*lower line*) pediatric cohorts. Mutations in *blue* indicate low plasma CFH level; mutations in *black* indicate normal plasma CFH level. *Ho* homozygous

**Fig. 3:**
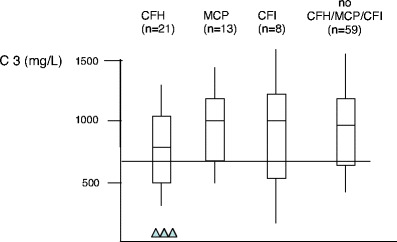
C3 plasma concentration in 101 children with aHUS (Italian and French pediatric cohorts), according to genetic subgroup. *Triangles* homozygous mutation; *horizontal line* represents 2SDs of normal

However, the concentration of CFH and the distribution of the molecular defect are more heterogeneous among pediatric patients. The results of *CFH* mutations in two independent cohorts of patients with pediatric onset aHUS, one from Italy (111 patients, 77 pedigrees) [32], and one from France (46 patients, 41 pedigrees) [33], are shown in Fig. 2. While 71% of *CFH* mutations were located in the exons encoding the SCR 18 to 20 in the Italian cohort, only 30% were located in SCR 20 in the French cohort, with another 30% in SCR 15 and other mutations scattered over the *CFH* gene. In addition, 70% of patients with *CFH* mutation in the French cohort presented with quantitative CFH deficiency [33], as opposed with approximately 20% in the Italian cohort [32]. When the two cohorts were mixed (Fig. 3), C3 levels were within the normal range in approximately 70% of patients. These data indicate that sequencing of *CFH* gene cannot be limited to SCR 18 to 20 in children with aHUS and that normal CFH and C3 level do not exclude a mutation in the *CFH* gene.

It is interesting to note that some mutations have been reported repeatedly in different studies [28]. All of them are located in the SCR20. For instance, the mutation Arg1210Cys has been reported in several unrelated aHUS patients from distinct geographic origins and with heterogeneous clinical phenotypes [34].

In addition to mutations in complement regulators, CFH autoantibodies leading to an acquired CFH functional deficiency have been reported in aHUS patients [20–22]. The binding epitopes of the autoantibodies were localized to the C-terminal recognition region of CFH, which represents a hot spot for aHUS mutations.

#### MCP (CD46)

**Fig. 4:**
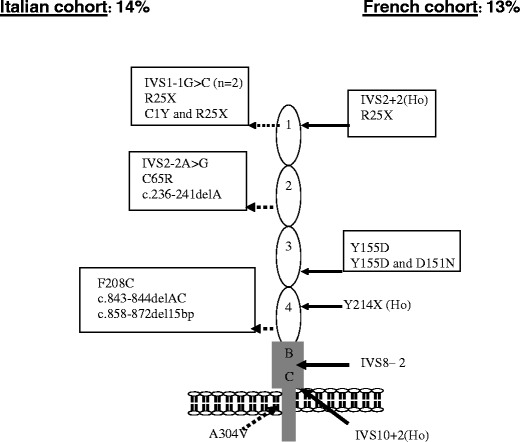
*MCP* mutations in the Italian (*left*) and French (*right*) pediatric cohorts. *Ho* homozygous

MCP is a widely expressed transmembrane glycoprotein that regulates complement activation by serving as a membrane-bound cofactor for the plasma serine protease CFI to cleave C3b (Fig. 1). Richards et al. have identified for the first time functionally significant *MCP* mutations in seven patients (three pedigrees) presenting a familial aHUS with a recessive form of inheritance [14]. More than 20 different mutations in *MCP* have now been identified in patients with aHUS [12, 13, 28]. Figure 4 shows the mutations identified in the Italian and French pediatric cohorts. Over 80% of the reported mutations caused a reduction in MCP expression, due to homozygous, compound heterozygous or heterozygous mutations. This deficiency of MCP leads to inadequate control of complement activation on endothelial cells after an initiating injury (Fig. 1). As indicated in Fig. 3, C3 levels in *MCP*-mutated patients were normal in the French cohort. Nevertheless, lower-than-normal C3 levels were observed in approximately one-third of Italian childhood (3/8) patients [32].

#### CFI

**Fig. 5:**
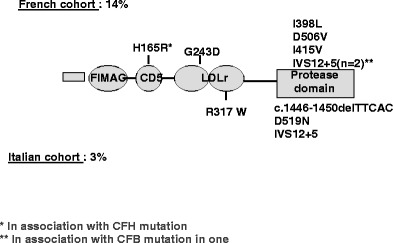
*CFI* mutations in the French (*upper line*) and Italian (*lower line*) pediatric cohorts

CFI is a two-chain serine protease in which the light chain carries the catalytic domain, while the heavy chain’s function is unclear. It down regulates the alternative complement pathway by cleaving the alpha chain of C3b in the presence of cofactor proteins (i.e. CFH and MCP). Fewer than 20 mutations in *CFI* have been reported in patients with aHUS, all heterozygous [28] (Fig. 5), and *CFI* mutations appear to be a less common cause of aHUS (between 5% and 10%) than are *CFH* and *MCP* mutations [15, 16, 32, 33]. CFI and C3 levels are most often normal [32, 33] (Fig. 3). However, the majority of *CFI* mutations induce a lack of protein synthesis, and only few mutations have been associated with a functional deficiency [17].

#### Recent identification of two new susceptibility factors, and the frequency of combined mutations

New complement genes associated with aHUS have recently been described. Goicoechea de Jorge et al. found a gain-of-function mutation (which increases C3bBb convertase stability) in *CFB*, associated with aHUS [18]. *CFB*-mutated patients exhibit permanent activation of the alternative pathway with low C3, while CFB plasma levels are normal. Mutations in *CFB* are rare, accounting for 0% to 3% of aHUS patients [28]. Approximately 10% of aHUS patients, without a mutation in *CFH*, *IF, MCP* or *CFB*, are found to have decreased serum levels of C3. Our group therefore sequenced *C3* coding exons in 30 such individuals from two independent cohorts (Newcastle and Paris) and identified ten heterozygous *C3* mutations [19].

It is important to note that at least 10% of patients have combined mutations, especially of *CFI* mutation with either *CFH* or *MCP* [32] or *CFB* or *C3* (unpublished data from the French Pediatric Registry).

In summary, aHUS is a disease where impairment of alternative pathway regulation leads to the excessive liberation of different cleavage fragments of C3, such as C3a and C5a, and to the formation of the C5b9 complex. These three components generate endothelial damage and microangiopathic lesions [35–38].

### Current diagnostic approach

In practice, in 2008, patients suspected of having aHUS are screened by the measurement of plasma complement C3, C4, CFH, CFI and CFB antigenic levels, membrane expression of MCP (CD46) in blood leukocytes, by the detection of CFH autoantibodies (Table 1) and by the genetic testing of at least the three now well-known susceptibility genes (*CFH*, *CFI*, and *MCP*) (Table 2). Plasma levels of the complement proteins and MCP expression according to mutation or the presence of anti-CFH autoantibodies are summarized in Table 3. Four considerations are important: (1) Assessment of plasma levels of complement proteins is insufficient, and genetic analyses are necessary for any patient with aHUS. (2) As mutations have been identified everywhere in the three genes, it is prudent for one to screen all exons of the genes, including *CFH*. (3) Since at least 10% of patients have mutations in two complement regulators, screening should be done to seek out mutations in all known predisposing genes. (4) The functional consequences of each genetic abnormality should be determined in vitro by mutagenesis. Our genetic screening strategy is more exhaustive than that recommended by Kavanagh et al. for adults [39], as we consider that all genes must be thoroughly screened in children, especially before transplantation.

**Table 1:** Assessment of plasma complement proteins and membrane complement proteins (*ELISA* enzyme-linked immunosorbent assay, *EDTA* ethylene diamine tetra-acetic acid)

Complement proteins	Plasma concentration (mg/l) (−2 to +2 SDs)	Technique	Laboratory test	Interpretation
C3	660–1,250	Nephelometry	Basic complement screen	Severe complement consumption through the alternative pathway is indicated by very low plasma levels of C3 and CFB. Frequently, there is only isolated low C3 level
CFB	93–380	Nephelometry	Specialized diagnostic	
CFH	330–680	ELISA	Specialized diagnostic	CFH or CFI less than 60% are compatible with quantitative deficiency
CFI	40–80	ELISA	Specialized diagnostic	
Anti-CFH autoantibody	Screening	ELISA	Specialized diagnostic	The titre is expressed in arbitrary units (AU)
MCP	Mean fluorescence intensity (MFI)	Flow cytometry analysis (FACS)^a^ with anti-MCP phycoerythrin (PE)-conjugated antibodies	Specialized diagnostic	No MCP expression is detected in patients with homozygous MCP deficiency. The MFI in patients with a heterozygous deficiency is around 50% of the normal range

^a^Usually performed on granulocytes or peripheral blood mononuclear cells in EDTA-blood samples

**Table 2:** Genetic screening (*MLPA* multiplex ligation dependent probe amplification, *N/A* not applicable)

Gene	Location	Method of choice for mutation screening	Number of exons	Frequency in aHUS (%)
*CFH*	RCA gene Chr 1	Direct sequencing analysis	22	15–30
*CFI*	Chr 4	Direct sequencing analysis	13	5–10
*MCP*	RCA gene Chr 1	Direct sequencing analysis	14	10–15
*C3*	Chr 19	Direct sequencing analysis	42	N/A
*CFB*	Chr 6	Direct sequencing analysis	18	2
*CFH-CFH R1* hybrid	RCA gene Chr 1	MLPA	Lack of exons 21 and 22 of SCR20 in *CFH* gene	N/A

**Table 3:** Summary of plasma levels of C3, C4, CFH, CFI and CFB and expression of MCP in the various subgroups. Very low C3 level is observed in only patients with homozygous or compound heterozygous *CFH* mutation and in *CFB*-mutated patients. In other situations, C3 level is generally mildly decreased or normal *N*

Gene	Protein level or expression					
	C4	C3	CFH	CFI	CFB	MCP
Mutation in *CFH*	N	Low or N	Low or N	N	Low or N	N
Mutation in *CFI*	N	Low or N	N	Low or N	Low or N	N
Mutation in *MCP*	N	N^a^	N	N	N	Low or N
Mutation in C*FB*	N	Low	N	N	N^b^	N
Mutation in *C3*	N	Low or N	N	N	Low or N	N
Anti-CFH-antibodies	N	Low or N	Low or N	N	Low or N	N

^a^Some *MCP*-mutated patients have low C3 levels ([32])

^b^Some *CFB*-mutated patients have low CFB levels (unpublished data from a patient of the French pediatric cohort)

Although investigation guidelines are out of the scope of this article, we point out that determination of the activity of ADAMTS (a disintegrin and metalloproteinase with thrombospondin motifs) 13 and screening for defective cobalamine metabolism are also mandatory in any child with aHUS [1, 3, 40]. The list of laboratories providing specialized investigations of the complement system and ADAMTS 13 is available at https://doi.org/espn.cardiff.ac.uk.

## Clinical characteristics of aHUS associated with genetic anomalies of complement proteins

In recent years, data from registries [32, 33, 41] and individual centers have allowed the analysis of clinical characteristics of aHUS in children according to the identified risk factors.

### Familial/sporadic aHUS, intrafamilial penetrance and genetic variability

**Fig. 6:**
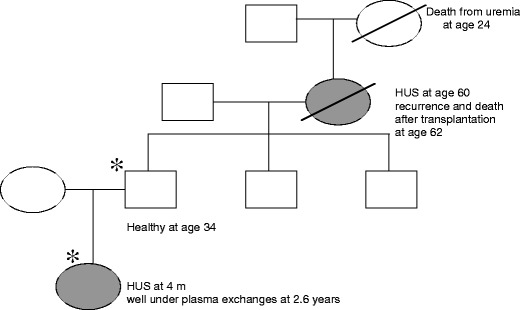
Intrafamilial phenotype variability in a family with heterozygous *CFH* mutation (W 1183 R, SCR 20). Ages are given in years. Affected individuals are indicated by *filled symbols*, deceased individuals by a *diagonal line*. Carriers of *CFH* mutation are indicated by *asterisks*. *m* months

Gender ratio is equilibrated in aHUS of pediatric onset [33]. The incidence of familial aHUS was 25% in the French Pediatric Registry [33] and 37% in the Italian Registry, which includes that in both children and adults [32]. The frequency of familial HUS is similar in the groups with *CFH*, *MCP* and *CFI* mutations and in the group with no mutation. Most frequently in familial aHUS, the disease occurs in siblings. However, in several families, the disease occurred in several different generations (Fig. 6). The absence of familial history of HUS does not preclude the possibility of a genetic transmission of the disease.

Penetrance of HUS associated with complement mutations has been shown to be approximately 50%. Only half of the family members who carry the mutation manifest the disease [38, 42, 43]. It has been suggested that polymorphisms of *CFH* and *MCP* influence the predisposition of an individual to aHUS and provide an explanation for the incomplete penetrance of the disease within families. In some families, it appeared that the proband had inherited the complement mutation from one parent and an allele carrying the polymorphism of *CFH* and/or MCP from the other parent, while the healthy mutation carriers did not inherit the aHUS-associated *CFH* and *MCP* polymorphisms [5, 34, 38, 42–44]. Another polymorphic variant of *CFH*-related genes (deletion of *CFH-R3* and *R1*) has been shown to be more frequent in aHUS patients than in controls [45].

In practice, it is impossible for one to forecast the risk of occurrence of HUS in family members presenting the mutation. Another problem is that several mutations may be present in one family, while some mutations are unknown. For example, in two families from the French Pediatric Registry, one child with aHUS had *CFH* or *CFI* + *C3* mutations, respectively, while a sibling also with aHUS had no mutation ([33] and unpublished data). This shows that other unidentified genetic risk factors may be present in the patient and in healthy family members.

### Age at onset

In the French pediatric cohort, patients harboring *CFH* or *CFI* mutations were markedly younger at disease onset (median age 6 months and 2 months, respectively) than patients with *MCP* mutation (median age 4 years 6 months) [33]. Noticeably, in the literature, the onset of the disease did not occur in any of the patients with *MCP* mutations before they were 1 year of age, except for one patient (patient 6 in [12], who probably had another mutation in addition to *MCP* mutation). Patients with no mutation in *CFH*, *MCP* or *CFI* may manifest HUS at any age (from 25 days to 15 years) [33]. In practice, onset before 3 months of age is highly suggestive of *CFH* or *IF* mutation.

### Triggering events

Another interesting point is the high frequency of an infectious triggering event. In the French and Italian pediatric cohorts, HUS onset followed an upper respiratory tract infection, fever, or diarrhea in 63% and 85% of patients from all subgroups, respectively. Interestingly, diarrhea preceded HUS in 13 (28%) patients from all subgroups, including 0157:H7 *E. coli*-associated bloody diarrhea in one child with an *MCP* mutation [33]. *MCP* mutation could be a risk factor of STEC-induced HUS, and possibly of severe forms, as a 4-year-old patient with an *MCP* mutation died from multi-visceral involvement after Stx-HUS [46]. Another observation, of an adult patient with *CFH* mutation and severe D+ HUS, has also been reported [47]. These observations show that the D+ or D− classification of HUS may be misleading, and that post-diarrheal onset does not exclude the possibility of genetic aHUS.

### Clinical course and outcome

The overall prognosis of aHUS in these genotyped cohorts is poor. Among the French pediatric cohort, four (8.6%) of the 46 children died and 11 (24%) developed ESRD after the first episode. Age at onset, familial or sporadic occurrence of HUS and C3 level were not predictive of outcome, while serum creatinine level at first flare was significantly associated with the outcome at 1 year [33]. Relapsing HUS may occur whatever the genotype as well as in patients with no identified mutations. However, the number of relapses is significantly more important in the *MCP*-mutated and in the non-mutated groups. Some of these patients have severe hemolytic anemia and thrombocytopenia during HUS episodes, with acute renal failure mostly due to hemoglobinuria (unpublished data from the French Pediatric Registry). Relapses with complete recovery is characteristic of patients with *MCP* mutations and some patients with no identified mutation. Extrarenal involvement during HUS episodes (ischemic manifestations in the central nervous system, multi-visceral manifestations of thrombotic microangiopathy) is not frequent (fewer than 10% in pediatric onset aHUS [33]). However, some observations suggest that complement dysregulation may be responsible for atheroma-like vascular complications. A child with a *CFH* mutation had an ophthalmologic ischemic/hemorrhagic complication after 3 years on dialysis [48], and a 10-year-old child with a mutation in *CFB* developed diffuse arterial stenosis, affecting cerebral, carotid, coronary and distal pulmonary arteries, celiac trunk and splenic artery (unpublished data from a patient in the French pediatric cohort).

The worst prognosis is in patients with *CFH* mutations. Among French children, 60% of those with *CFH* mutation had either died or had ESRD by 1 year after onset (including 40% by as soon as the first episode) in comparison with 50% of the *CFI*-mutated patients, 0% of the *MCP*-mutated patients and 32% in the unexplained group. At 5 years after onset, the percentage of patients with ESRD was 73%, 50%, 38% and 32% in the *CFH*, *CFI*, *MCP* and unexplained groups, respectively [33]. Similar data were derived from the Italian cohort of pediatric patients, with, respectively, 68%, 67%, 10% and 28% of patients developing ESRD in the long term [32]. It therefore appears that patients with *CFH* mutation are at the highest risk for developing ESRD very early during the course of the disease, while patients with *CFI* mutation and patients with no mutation either develop ESRD during the year of onset (50% and 30% risk, respectively) or will do well during subsequent years, some of them (mainly unexplained aHUS) with a relapsing course but preserved renal function. *MCP*-mutated patients have a different course: renal function is generally preserved in the first year(s) of the disease, but approximately 30% of patients will reach ESRD after a relapsing course over several years [33].

In practice, it is clearly helpful for us to know which complement mutation each individual patient has, so that the outcome may be predicted and adequate therapeutic decisions made.

## aHUS associated with anti-CFH antibodies

The subgroup of aHUS associated with anti-CFH autoantibodies accounted for 6% of aHUS in the French pediatric cohort [20] to 11% in the German cohort, including adults and children [21, 22]. Clinical symptoms are not specific, except that the disease usually presents when the child is between 3 years and 14 years of age. C3 level is generally slightly decreased, and CFH level in usually normal [20] (Table 3). Interestingly, most patients with anti-CFH antibodies are homozygous for deletion of the *CFHR1* and *CFHR3* genes [22]. Consideration of the therapeutic implications (plasma exchanges, glucocorticoids and immunosuppressive treatment), screening for anti-CFH antibodies in any child with aHUS, is mandatory.

## Treatment

### Plasmatherapy

There are many reports on plasmatherapy in aHUS, but there are no controlled trials. These plasma-based therapies, although evidence is lacking, remain the mainstay of treatment for aHUS [49]. Fresh frozen plasma (FFP) replaces defective CFH, CFI, CFB and C3. Plasma exchange (PE) removes mutated CFH, CFI, CFB and C3, anti-CFH antibodies and other triggers of endothelial dysfunction, while restitution with FFP restores the functional proteins. In addition, PE prevents volume overload and cardiac failure when large amounts of FFP are infused.

Logically, the few patients with a complete quantitative deficiency of a complement protein such as CFH might do well simply with FFP infusions that provide the normal protein. However, the majority of patients have functional deficiency of one or several complement proteins. These mutant proteins are present in the circulating blood but also on cell surfaces, where they interfere with the protective function of the normal protein. PE could be necessary to withdraw these mutant proteins.

#### Plasmatherapy in patients with CFH mutation

A limited number of case reports suggest that plasmatherapy can be effective to rescue and prevent aHUS flares in *CFH*-mutated patients. As indicated in Table 4, four patients with complete quantitative CFH deficiency benefited from FFP infusions [50–54]. In most of them, treatment was started when serum creatinine level was normal or only slightly increased. Another point is that follow-up under plasmatherapy was only 7 months to 4 years. The patient of Nathanson et al. was well for 4 years under weekly FFP infusions [51]. Nevertheless, relapse of HUS when the patient was aged 8 years was resistant to daily PE [52].

**Table 4:** Results of plasmatherapy in four aHUS patients with CFH quantitative deficiency secondary to homozygous or compound heterozygous *CFH* mutations. FFP infusions may suffice, but follow-up is short and secondary failure happened in one patient. *Ho* homozygous, *He* heterozygous, *FFP* fresh frozen plasma infusions, *PE* plasma exchange with FFP for restitution, *m* months

		During HUS episodes		Preventive plasmatherapy	
Author (reference)	Number of patients	Failure	Success	Failure	Success (follow up)
Landau et al. [50]	1 (Ho)		FFP 10 ml/kg	FFP 10 ml/kg	FFP 12–20 ml/kg twice weekly (7 m)
Nathanson et al. [51]	1 (Ho)	Secondary resistance to PE 60 ml/kg × 11 days			FFP 15 ml/kg weekly (4 years)
[52]					
Licht et al. [53]	1 (Ho)				FFP 20 ml/kg every 2 weeks (18 months)
Cho et al. [54]	1 (compound He)		FFP 15 ml/kg × 3/week	No prevention 4 relapses over 17 months	

Reports on plasmatherapy in five patients with CFH functional deficiency suggest that intensive plasmatherapy might also be beneficial in this situation [55–58] (Table 5). In a report by Davin et al. [58], two twin sisters were successfully treated by PE with FFP, 40 ml/kg daily for 10 days, at their first episode of HUS. One twin subsequently received FFP infusions only during recurrences of thrombocytopenia, but she progressed to ESRD after 4 months. The other twin was maintained on PE every 2 weeks, which was intensified to daily PE during two relapses. Serum creatinine level was normal, and no relapses occurred during 3.6 years of follow-up, suggesting that PE therapy has benefits over FFP infusions [58]. Again, follow-up in these five patients was only 1 year to 3.6 years, and it is not certain if the favorable effect of plasmatherapy can be maintained for decades.

**Table 5:** Results of plasmatherapy in five aHUS patients with CFH functional deficiency, secondary to heterozygous *CFH* mutations. Large amounts of FFP are necessary, and PE appears to have advantages over FFP infusions. *FFP* fresh frozen plasma infusions, *PE* plasma exchange with FFP for restitution, *ESRD* end-stage renal disease

		During HUS episodes		Preventive plasmatherapy	
Author (reference)	Number of patients	Failure	Success (follow up)	Failure	Success (follow up)
Stratton and Warwicker [55]	1 (adult)		PE ≃ 40 ml/kg daily × 1 week, tapered to weekly × 3 months then FFP ≃ 15 ml/kg weekly × 1 month (1 year)		
Gerber et al. [56]	1				PE weekly × 4 months, then FFP 20 ml/kg (3 years)
Filler et al. [57]	1			FFP 30 ml/kg weekly	FFP 30 ml/kg twice weekly or 40–45 ml/kg weekly
					→ proteins 92 g/l
					→ PE 40–45 ml/kg weekly or every 4–5 weeks with FFP infusions in between (1 year)
Davin et al. [58]	1st twin	FFP 10 ml/kg daily (during relapses)	PE 40 ml/kg daily × 10 days (1st episode)	No prevention → ESRD at 4 months	
	2nd twin		PE 40 ml/kg daily × 10 days		PE 40 ml/kg every 2 weeks (3.6 years)

#### Plasmatherapy in patients with CFI, MCP or CFB mutation

As MCP is not a circulating protein, a beneficial effect of plasmatherapy is unlikely to be expected in patients with a mutation in *MCP*. Published data indicate that 70–80% of patients undergo remission from acute episodes, whether or not they have been treated with plasma [32]. Nevertheless, a potential benefit of PE during relapses, to clear up noxious entities such as aggregating factors/cytokines, remains possible.

The results of plasmatherapy in patients with the *CFI* and *CFB* mutations are not well documented [16, 18, 59]. One may suspect that patients with a mutation in *CFB* need large amounts of FFP and very frequent PE to overcome the overproduction and resistance to decay of the alternative pathway C3 convertase induced by the gain-of-function *CFB* mutation. On the other hand, one could also speculate that supplementation in C3 might, in fact, be deleterious by bringing substrate to the unregulated activation of the complement cascade.

#### Plasmatherapy in practice: when to start? How much? How frequently? How long?

Recommendations can only be empirical. Recent guidelines from the European Paediatric Study Group for HUS [40] recommend that plasmatherapy be started as early as possible, within 24 h of presentation, in parallel with conservative treatment (dialysis, transfusion, antihypertensive treatment etc.). First line treatment should be PE, with exchange of 1.5 plasma volume (60–75 ml/kg) per session, replaced by FFP. When PE cannot be performed within 24 h of presentation, plasma infusions of 10–20 ml/kg should be given if the patient is not volume overloaded and/or hypertensive. PE should be daily for 5 days, then five times a week for 2 weeks and then three times a week for 2 weeks [40]. Further frequency of PE would be predicted on an individual basis.

### Prevention of infections, and vaccinations

Considering the frequency of relapses triggered by infections, eradication of adenoidal, tonsil and dental infections is warranted. It is also justifiable that preventive plasmatherapy be intensified during infections (or at least biological monitoring be intensified). A triggering effect from vaccinations is possible, although rarely documented. Probably, the beneficial effect of vaccination outweighs its risk, as in other immunity diseases. The few patients with permanent activation of the complement alternative pathway and very low C3 levels (patients with *CFB* mutation or homozygous or compound heterozygous *CFH* mutations) have to be considered as immunodeficient. These patients must receive permanent preventive antibiotic therapy (penicillin or macrolides in case of allergy to B-lactamines) as well as vaccination against *Neisseria meningitis* and *Streptococcus pneumoniae* [29].

### Future treatments

#### CFH concentrate

A human plasma-derived CFH concentrate is being developed by the Laboratoire Français du Fractionnement et des Biotechnologies, and it received the European orphan drug designation in January 2007. This will be an easier option for patients with *CFH* mutation, although the respective place of PE and CFH substitution will have to be carefully thought about in patients with a functional CFH deficiency.

#### Complement inhibitors

Monoclonal humanized antibodies against key activating components of complement such as C5 should be beneficial, by decreasing the damage mediated by the anaphylatoxin C5a and preventing the formation of the membrane attack complex on cell surfaces. Prevention of C5 activation has been shown to ameliorate glomerulonephritis in CFH −/− mice [60]. The long-term efficacy and tolerance of the anti-C5 monoclonal antibody eculizumab has been demonstrated in large cohorts of patients with paroxysmal nocturnal hemoglobinuria [61, 62].

### Treatment of patients with anti-CFH antibodies

PE, logically, should be considered as first line treatment for patients with anti-CFH antibodies. Steroids and various immunosuppressive treatments, including rituximab, all tried empirically, should be administered to prevent the production of antibodies after PE cessation [20, 21, 63].

## Transplantation

### Post-transplantation recurrence of HUS and thrombosis

The post-transplantation course has been analyzed in approximately 80 patients with aHUS screened for *CFH*, *CFI* and *MCP* mutations, including children [33] or both pediatric and adult onset aHUS [32, 64]. In the French pediatric series, of the 24 renal transplantations performed in 15 aHUS children, 16 (67%) failed. Of the 16 graft failures, eight (50%) were due to graft vascular thrombosis and five (31%) to recurrence of HUS [33]. The high proportion of vascular thrombosis was most likely related to the thrombogenic role of complement dysregulation.

A recent review of the literature indicated that, out of 34 patients with *CFH* mutations, 26 (76%) had post-transplantation recurrence, and 21 of those (81%) lost the graft within a year after recurrence [24]. Of eight patients with *CFI* mutations, seven (88%) had recurrence, and all lost their grafts due to recurrence within the year. On the other hand, as non-mutated MCP is brought by the graft, no post-transplantation recurrence is expected to occur in *MCP*-mutated patients. Nevertheless, two of ten *MCP*-mutated patients had post-transplantation recurrence. One of them most probably had another complement anomaly, suggested by biological signs of complement activation [12]. In the other patient, endothelial microchimerism was suggested by the colonization of the graft endothelia by the recipient’s cells [65]. The risk of recurrence in *CFB*- and *C3*-mutated patients is not well documented. The risk of recurrence in patients with no *CFH*, *CFI* or *MCP* mutation is 30% [24]. Finally, the risk of recurrence in patients with anti-CFH antibodies may be expected to be important if a high titer of antibodies persists at the time of transplantation.

### Kidney donation by living related donors is contraindicated

Considering the risk of graft loss due to recurrence, living-related kidney donation must be considered as contraindicated for patients with *CFH*, *CFI*, *CFB* and *C3* mutation and questionable for patients with unexplained aHUS. Living related kidney donation is debatable for patients with *MCP* mutation. In addition, the risk that the donor might develop HUS after kidney donation has to be taken into account. This has been reported in four donors aged 21–31 years, who had HUS 3 weeks to 10 months after donation [24, 66]. *CFH* mutation was subsequently demonstrated in one of the recipients and his donor. Considering the incomplete penetrance of the disease, the role of complement gene polymorphisms and the genetic variability within members of a single family, it is impossible to reach a 100% certitude of “no risk” for living related donors. As indicated above, among siblings with aHUS, it may be that some have mutations in the complement system, while some have no mutation identified, which indicates that other unidentified risk factors are present in the family, including the potential donor.

### Can post-transplantation aHUS recurrence be prevented?

Bilateral nephrectomy of native kidneys is often performed before transplantation because of severe hypertension or ongoing hemolysis and thrombocytopenia. Unfortunately, it does not appear to reduce the risk of recurrence of aHUS after transplantation [24]. Avoidance of calcineurin inhibitors also is not associated with a reduced incidence of HUS recurrence.

#### Plasmatherapy

Many genotyped patients of historical series have received some form of plasmatherapy at the time of recurrence [33, 64]. As time at plasmatherapy initiation, modalities (PE or FFP infusions), volume of FFP infused or exchanged, frequency and duration were highly variable, the effect of treatment is difficult to ascertain. Nevertheless, the efficiency of intensive prophylactic plasmatherapy started before renal transplantation seems to have been demonstrated in one family [58, 67]. Three children of this family had aHUS with *CFH* heterozygous S1191L mutation, SCR20. The eldest child lost two grafts due to recurrent HUS. One of the identical twins had preserved renal function with prophylactic PE therapy (see above). The second twin had renal transplantation with PE performed just before surgery and maintained after surgery, 40 ml/kg FFP, daily for 7 days, then every 2 weeks. There were two recurrences of HUS during cytomegalovirus infection, which were efficiently treated by daily PE. Weekly PE was maintained subsequently, and serum creatinine was 120 µmol/l 5 years 8 months after transplantation ([67], and personal communication J.C. Davin, 2008).

Three patients with *CFI* mutations lost their grafts, despite having PE, but none of them was treated prophylactically [16, 59].

#### Combined liver and kidney transplantation

As CFH is synthesized in the liver, liver transplantation has been proposed for patients with severe forms of HUS and *CFH* mutation. The first three combined liver–kidney [2] and auxiliary liver [1] transplantations were disappointing, as one child had severe neurologic sequelae [68] and two children died [69, 70] (Table 6). In one child who died from primary liver non-function, diffuse hepatic thrombotic and ischemic lesions were observed, most likely due to the thrombogenic effect of complement activation products deposited on liver vessels after transplantation [70]. These initial experiences suggested that liver transplantation, known to trigger complement activation, ought to be performed under intensive plasmatherapy, to correct the complement dysregulation before and during the operative period. The first successful combined liver and kidney transplantation in Mount Sinai Hospital, New York, USA, was reported in 2006 by Saland et al. [71] in a 5.6-year-old child who had lost a first kidney graft due to recurrence and had received one PE with FFP just before surgery and FFP infusion during surgery; plasma therapy was stopped thereafter. Enoxaparin and aspirin were administrated post-operatively. There was no HUS recurrence, and both grafts had excellent function at a 4 year follow-up examination ([71], and personal communication J. Saland, 2008). Another child in New York and two children in Helsinki [72] have undergone combined liver and kidney transplantation with similar protocols, with no recurrence of HUS and excellent function of both grafts during 8 months to 1.5 years of follow-up (Table 6).

**Table 6:** Results of liver or combined liver and kidney transplantation in seven children with *CFH* mutations. Pre- and intra-operative intensive plasmatherapy appear as a prerequisite for success. *PTLD* post-transplant lymphoproliferative disease

Authors (reference)	Age of patient (years)	*CFH* mutation	Transplantation	Plasmatherapy		Post-operative anticoagulation	Outcome (follow-up)
				Pre-operative	Per-operative		
Remuzzi et al. [68]	2	W 1183 R	Liver + kidney	No	No	No	Liver failure at day 26; re-transplantation neurologic sequels
		SCR 20					
Cheong et al. [69]	2.5	C926 F	Auxiliary liver	No	No	No	Death at 10 months from infections and PTLD
		SCR 15					
Remuzzi et al. [70]	2	E 1172 stop	Liver + kidney	No	No	No	Primary non-function of liver, thrombotic/ischemic lesions, post-operative death)
		SCR 20					
Saland et al. ([71] and personal communication)	2.2	C 973 Y, SCR 15, V 1197 A, SCR 20	Liver + kidney	PE 50 ml/kg	FFP 19 ml/kg	Yes	Both grafts successful (4 years)
	4	S 1191 L, SCR 20	Liver + kidney	Idem	Idem	Yes	Both grafts successful (1.5 years)
Jalanko et al. [72]	1.6	R 1215 Q	Liver + kidney	PE 98 ml/kg	FFP 36 ml/kg	Yes	Both grafts successful (15 months)
		SCR 20					
	16	R 1215 Q	Liver + kidney	PE 52 ml/kg at H-12 and 86 ml/kg pre-op	PE 70 ml/kg	Yes	Both grafts successful (8 months)
		SCR 20					

In summary, combined liver–kidney transplantation covered by intensive peri-transplant plasmatherapy started before the operation now appears as a reasonable therapeutic option for patients with mutations in *CFH* and, possibly, for patients with mutations in another factor synthesized in the liver, CFI. The possibility to extend the procedure to patients with *CFB* and *C3* mutation is complicated by the not negligible extrahepatic synthesis of these compounds. Nevertheless, the decision between kidney transplantation (with pre- and peri-operative plasmatherapy, subsequently maintained life-long) and combined liver and kidney transplantation (with pre- and intra-operative plasmatherapy) has to be taken on an individual basis. The choice of combined transplantation is logical if the patient or a family member with the same mutation has lost a graft due to recurrence. In all other situations, careful evaluation of the potential risks and benefits is needed. In particular, the decision of liver transplantation in patients with a *CFH* mutation and preserved renal function is difficult. The alternative of CFH concentrate infusions or anti-C5 monoclonal antibodies might be an easier option in the future.

## Conclusion and recommendations in 2008

1. Determination of C3, CFH, CFI and CFB levels, expression of MCP and screening for anti-CFH antibodies is indicated for all patients with aHUS. Normal C3 level does not eliminate the presence of *CFH* or *CFI* mutation or of anti-CFH antibodies.
2. Genotyping of *CFH*, *CFI* and *MCP*, and if possible *CFB* and *C3*, is indicated for all patients with aHUS, even if plasma levels are normal.
3. The identified mutation has to be regarded as a risk factor for HUS, not as the direct cause. The association of mutations in several genes is not exceptional. Penetrance of the disease is 50% in patients with a mutation in complement. Therefore, the risk of developing HUS is difficult to predict in family members with the mutation.
  Intrafamilial genetic heterogeneity exists, suggesting that unknown genetic factors are present.
4. A post-diarrheal onset of HUS can be observed in all groups. Therefore, genotyping must be performed for patients with uncertain diagnosis of D + /STEC + HUS, especially before transplantation.
  The worst prognosis is in patients with *CFH* mutation, who are at high risk of ESRD as soon as at first flare or within the year of onset.
5. Plasmatherapy (PE with FFP) should be started as early as possible. Although evidence is lacking, benefit is expected mainly in *CFH*-mutated patients and in patients with anti-CFH antibodies. Benefit is likely in all other subgroups of aHUS, except the *MCP* subgroup, where spontaneous remission generally occurs.
6. The risk of graft loss due to HUS recurrence or graft thrombosis is high in patients with *CFH* and *CFI* mutations, while it is very low in patients with *MCP* mutations.
  Family living donor transplantation is contraindicated, because of the risk of graft loss due to recurrence and the risk that donors themselves might have HUS after donation, due to unknown genetic factors shared with the recipient.
  Kidney transplantation under pre-, intra- and post-operative intensive plasmatherapy may be successful in some patients.
  Combined liver and kidney transplantation under pre- and intra-operative plasmatherapy, and post-operative anticoagulation, has been successful in a few patients with *CFH* mutation. This option will now have to be considered on an individual basis for patients with mutations in other factors synthesized in the liver.
7. Hope for the future relies on therapies which could prevent ESRD, such as CFH concentrate or anti-C5 monoclonal antibodies.

## Multiple choice questions

(Answers appear after the reference list)

1. A 4-year-old girl has aHUS (no diarrhea, results of test for Stx negative in stools, hemoglobin 8 g/dl, schizocytes 3%, thrombocytes 60,000/mm^3^, serum creatinine 3.4 mg/dl). Which biological investigations do you prescribe at admission?
  - C3
  - Anti-CFH antibodies
  - C3, C4, CFH, CFI, MCP, anti-CFH antibodies, ADAMTS 13
  - ADAMTS 13
2. A 2-month-old boy is admitted for aHUS (hemoglobin 6 g/dl, schizocytes 6%, thrombocytes 40,000/mm^3^, serum creatinine 2.4 mg/dl). C3 is 520 mg/l, CFH and CFI levels are normal. Which of the complement genes would you recommend be screened first for mutation?
  - None
  - *MCP* only
  - *CFH* and *CFI*
  - *CFI* only
3. A 6-year-old girl has aHUS with hemoglobin 5 g/dl, schizocytes 4%, thrombocytes 35,000/mm^3^, port wine colored urine with 100,000 red blood cells (RBCs)/ml, serum creatinine 2 mg/dl. She has already had one similar episode, when she was 2 years old. C3 and ADAMTS 13 levels were normal. Among complement anomalies, which is the most likely?
  - *CFH* mutation
  - *MCP* mutation
  - Anti-CFH antibodies
  - *CFB* mutation
4. A 2-year-old child is admitted for a first episode of non-post-diarrheal aHUS. The child is anuric, has gained 1.8 kg body weight (BW) within 1 week, and his blood pressure (BP) is 135/90 mmHg. Hemoglobin level is 6 g/dl; there are schizocytes 6%, and the platelet count is 80,000/mm^3^. Serum creatinine is 6 mg/dl and serum potassium 6 mmol/l. His grandmother had died of HUS at the age of 32 years. How will you proceed with treatment?
  - Dialysis to correct volume overload and hyperkalemia + RBC transfusion. No subsequent plasmatherapy
  - Plasma exchange first
  - FFP infusion first
  - Dialysis + RBC transfusion, to correct BP, hydro-electrolytic disequilibrium and anemia. Then (i.e. within 24 h) plasma exchange with FFP for restitution.
